# Supplementary material for: Uncovering natural variation in root system architecture and growth dynamics using a robotics-assisted phenomics platform
Source: eLife. 2022 Sep 1;11:e76968. doi: 10.7554/eLife.76968 (PMC9499532; doi:10.7554/eLife.76968)
Supplement: Figure 6—source data 1. — (A) Manhattan plots of genome-wide association (GWA) run on each of the nine RSA traits at each time point with corresponding Q-Q plots (B). SNPs highlighted in black pass the threshold, gray indicates SNPs in linkage disequilibrium (LD). Genes in this region are reported in Supplementary file 4. (C) SNP development of the 29 unique SNPs that pass the Bonferroni threshold at least once throughout the time series. [file elife-76968-fig6-data1.pdf]

Average angle

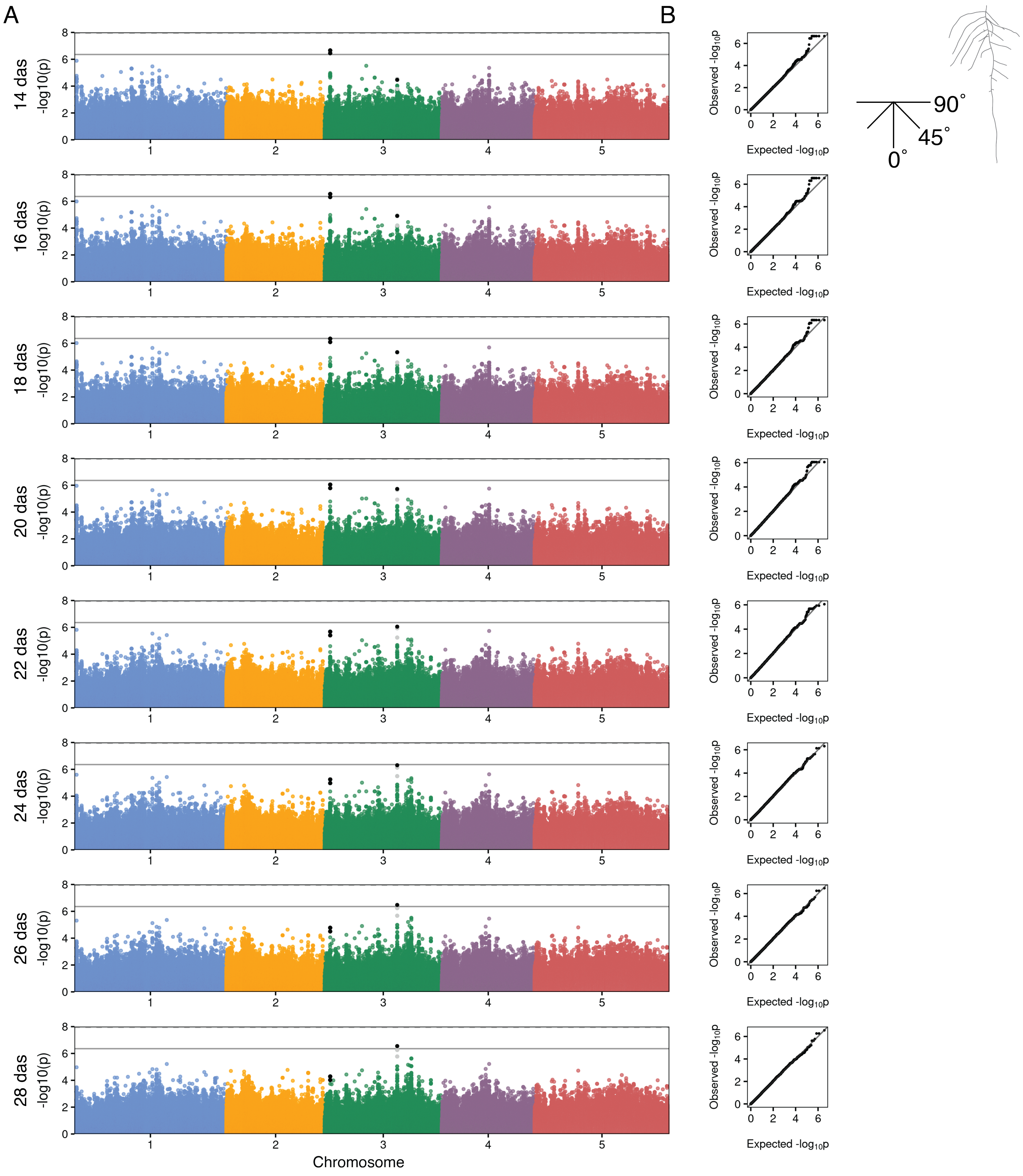

Average angle per day

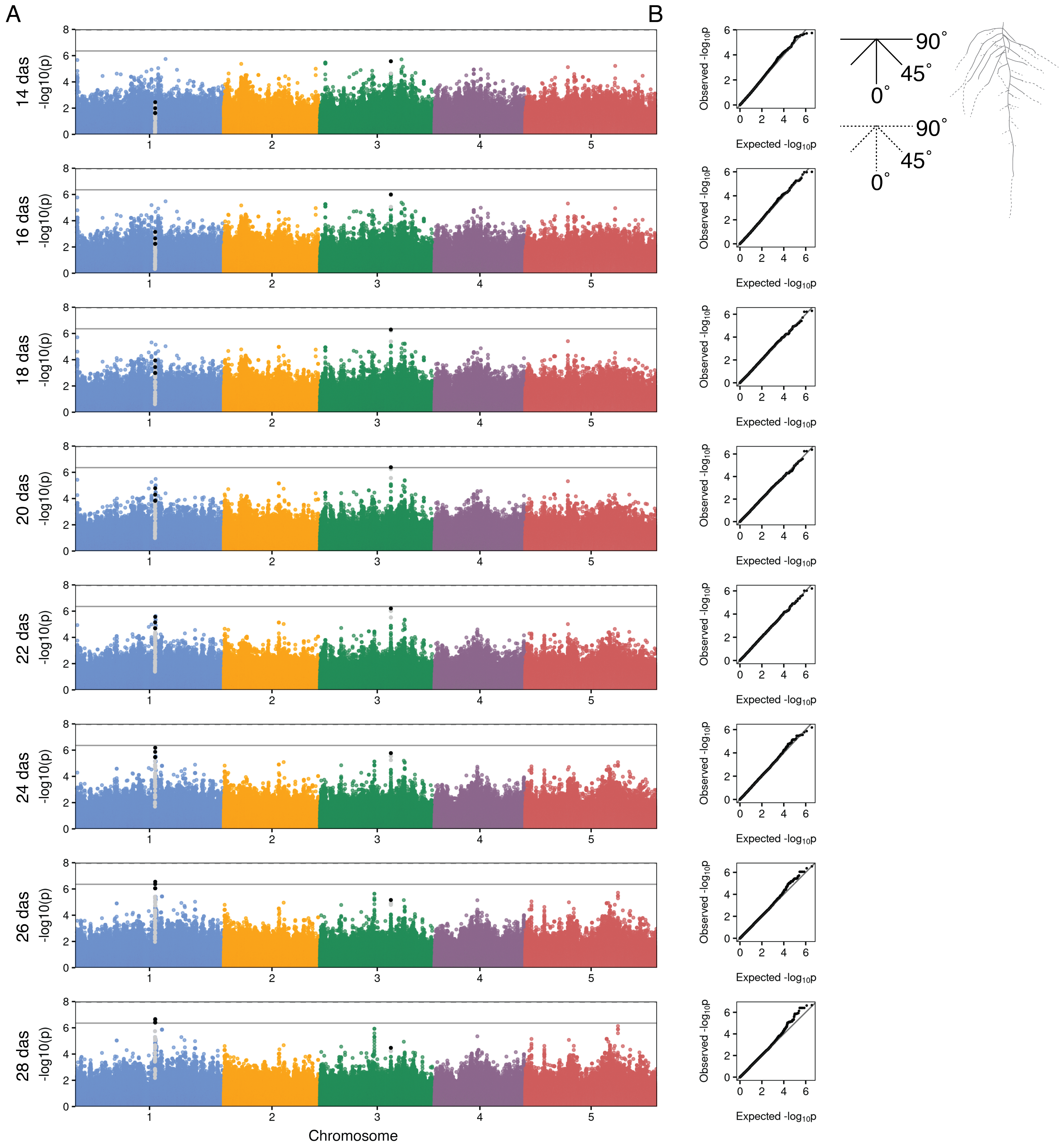

Convexhull

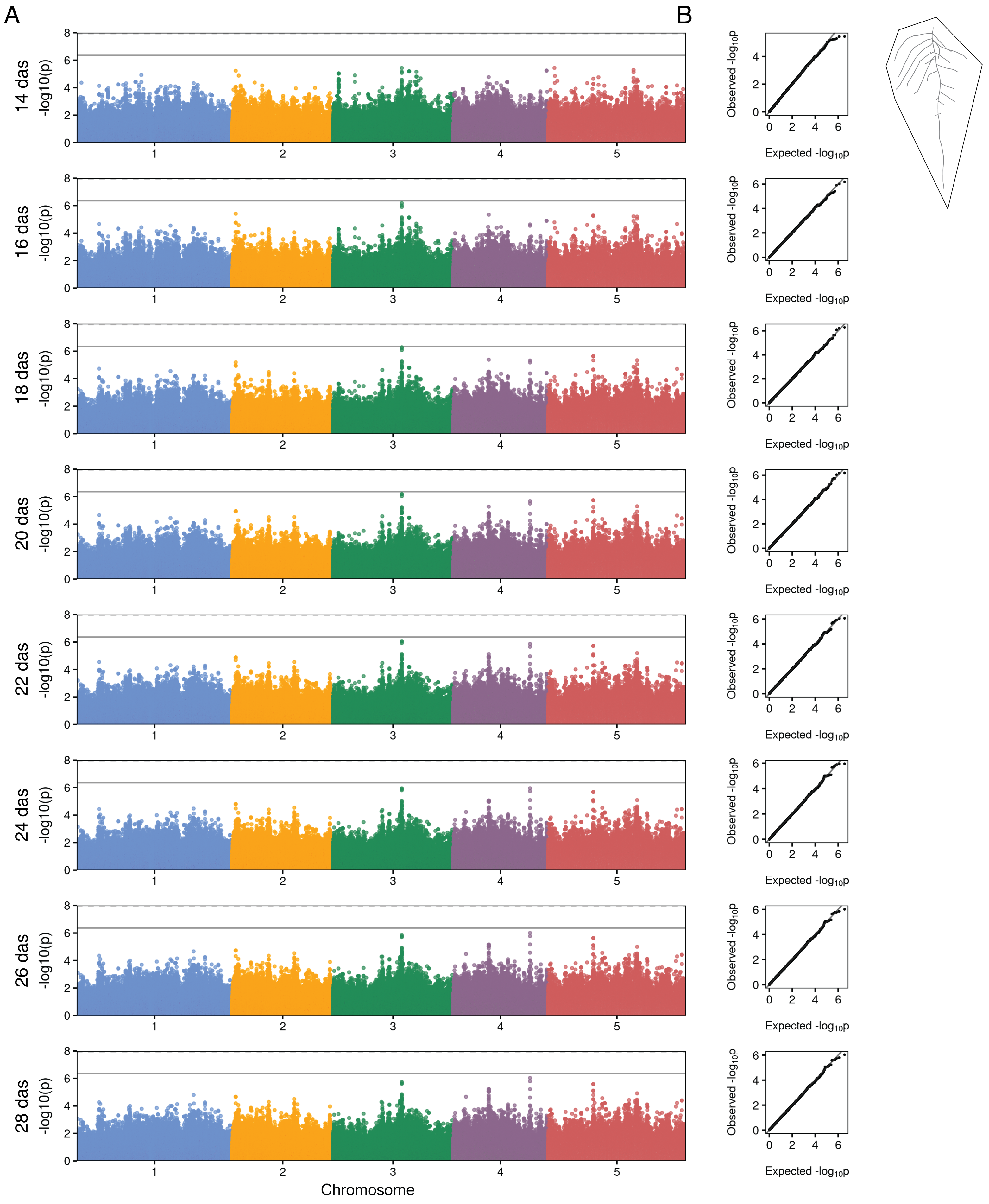
A
B
14 das
16 das
18 das
20 das
22 das
24 das
26 das
28 das
-log10(p)
Chromosome
1
2
3
4
5
Observed -log10p
Expected -log10p

Depth

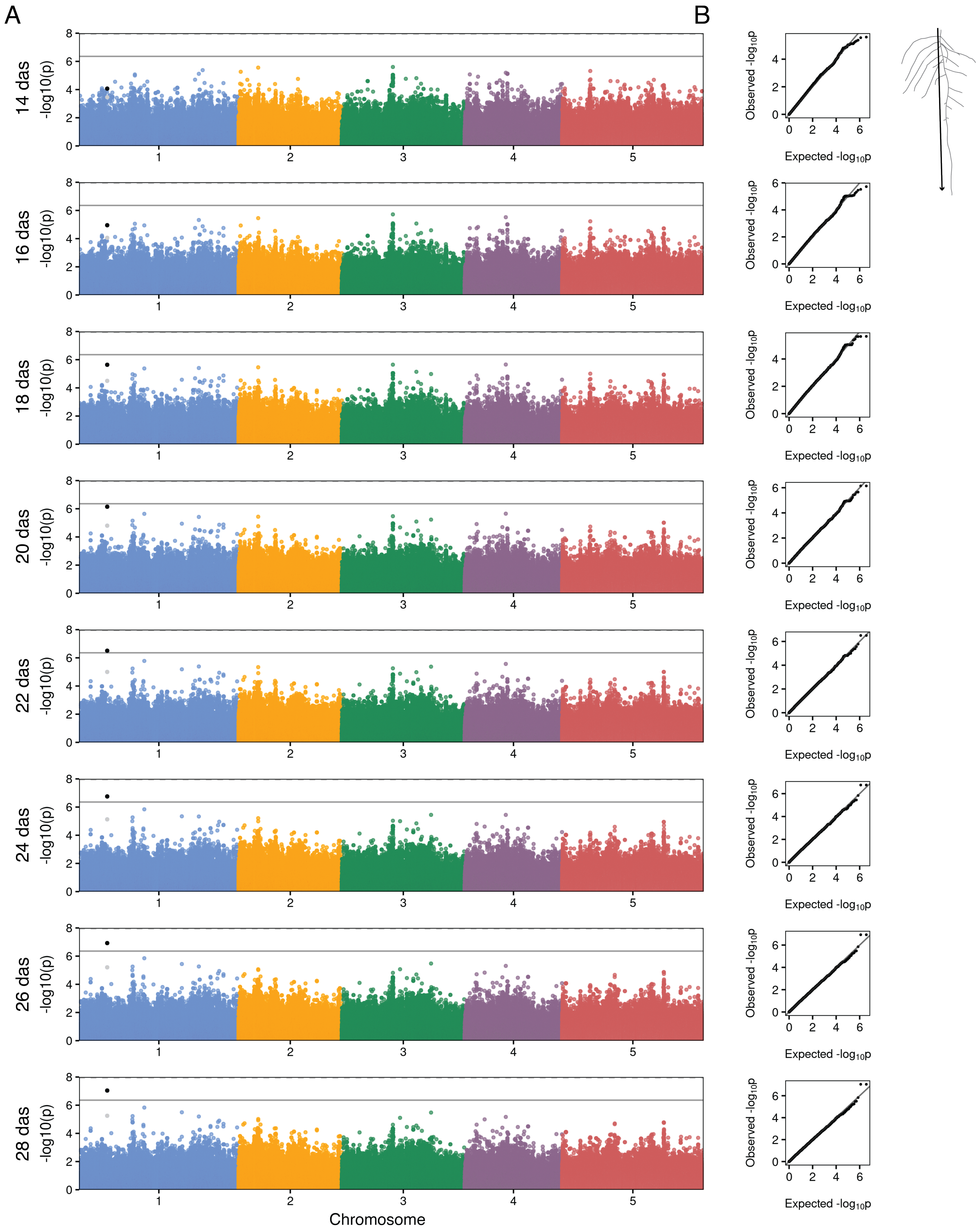

A
B
14 das
16 das
18 das
20 das
22 das
24 das
26 das
28 das
-log10(p)
Observed -log10p
Expected -log10p
Chromosome
1
2
3
4
5

Depth to width ratio

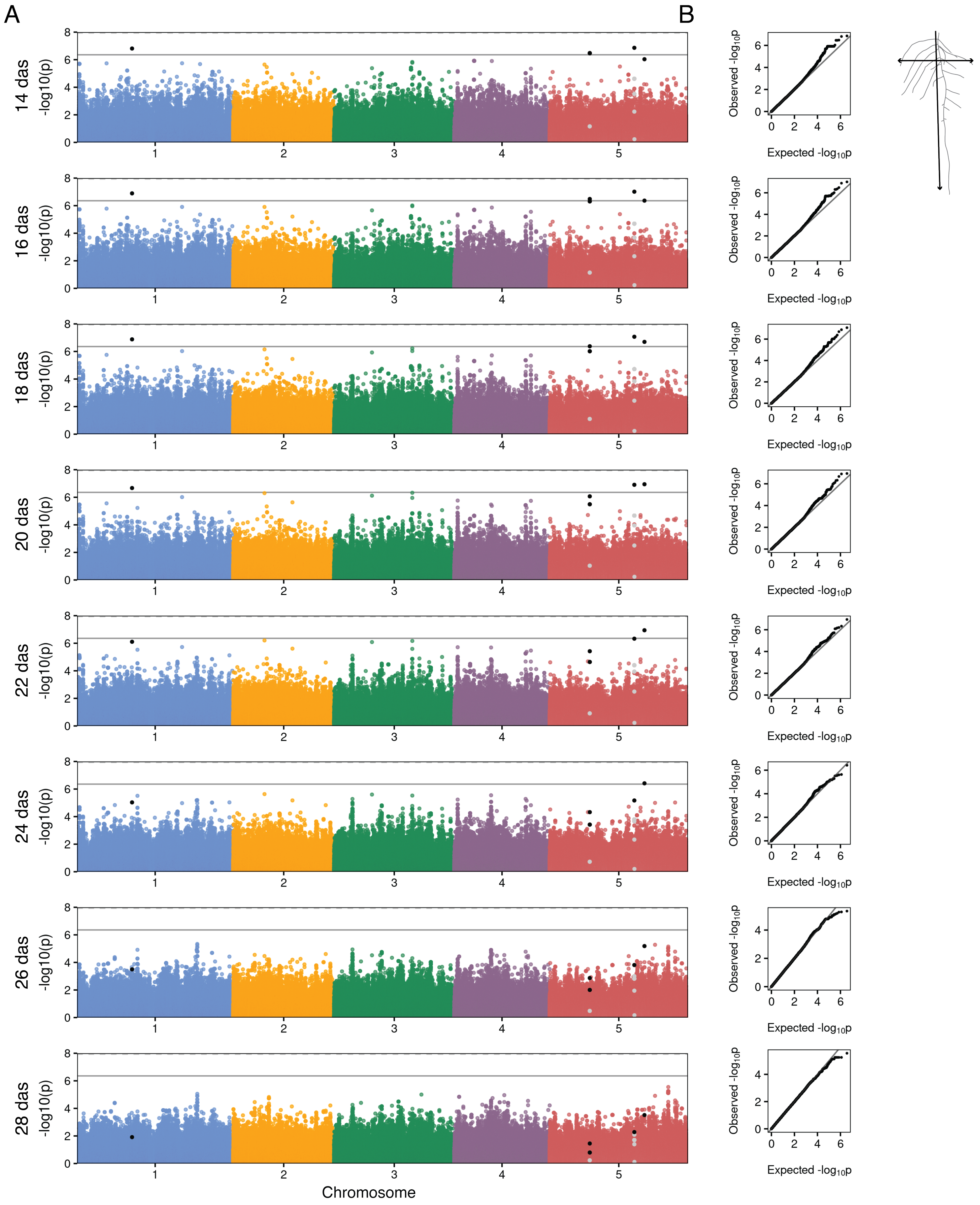

Length per day

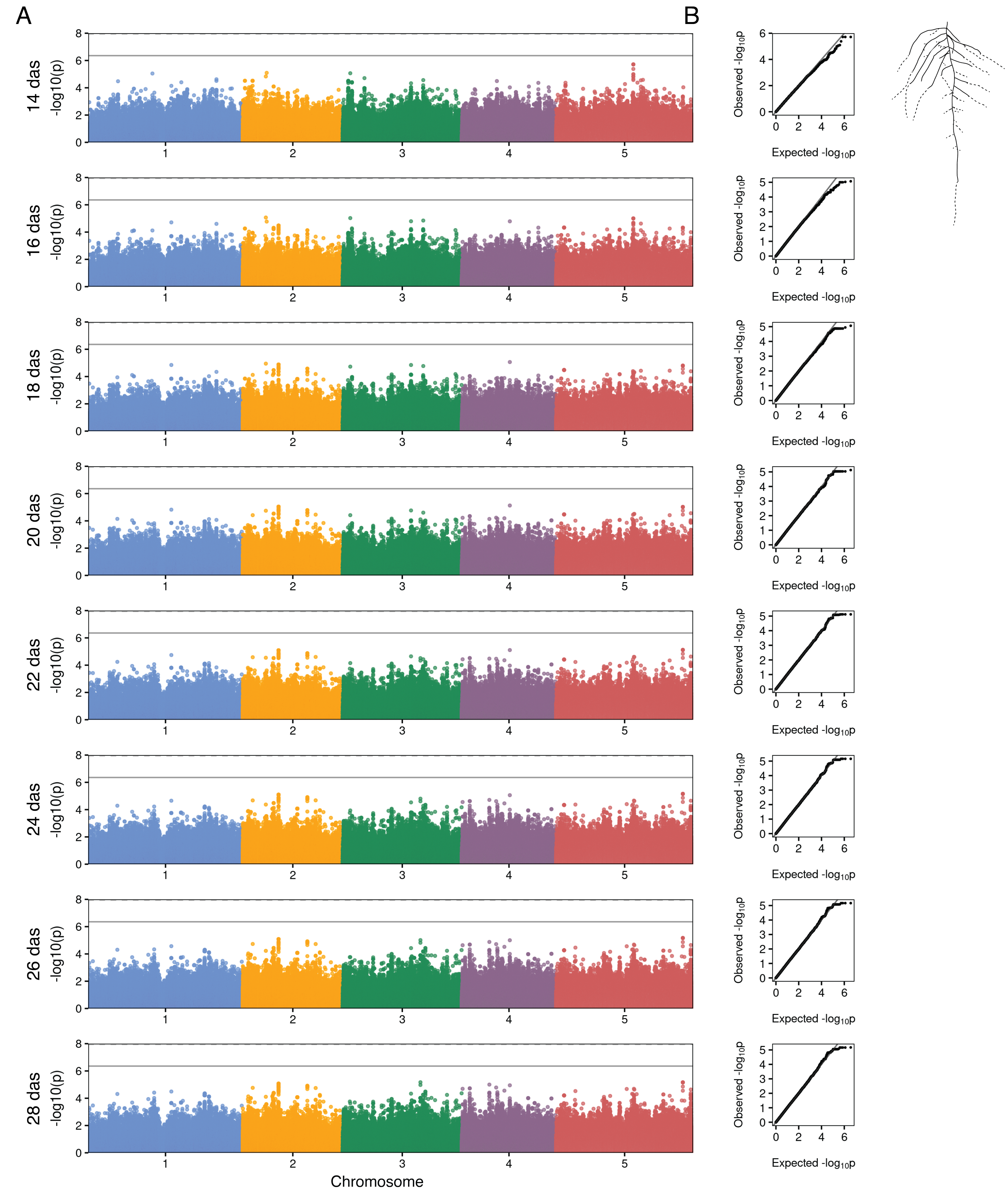

Total length

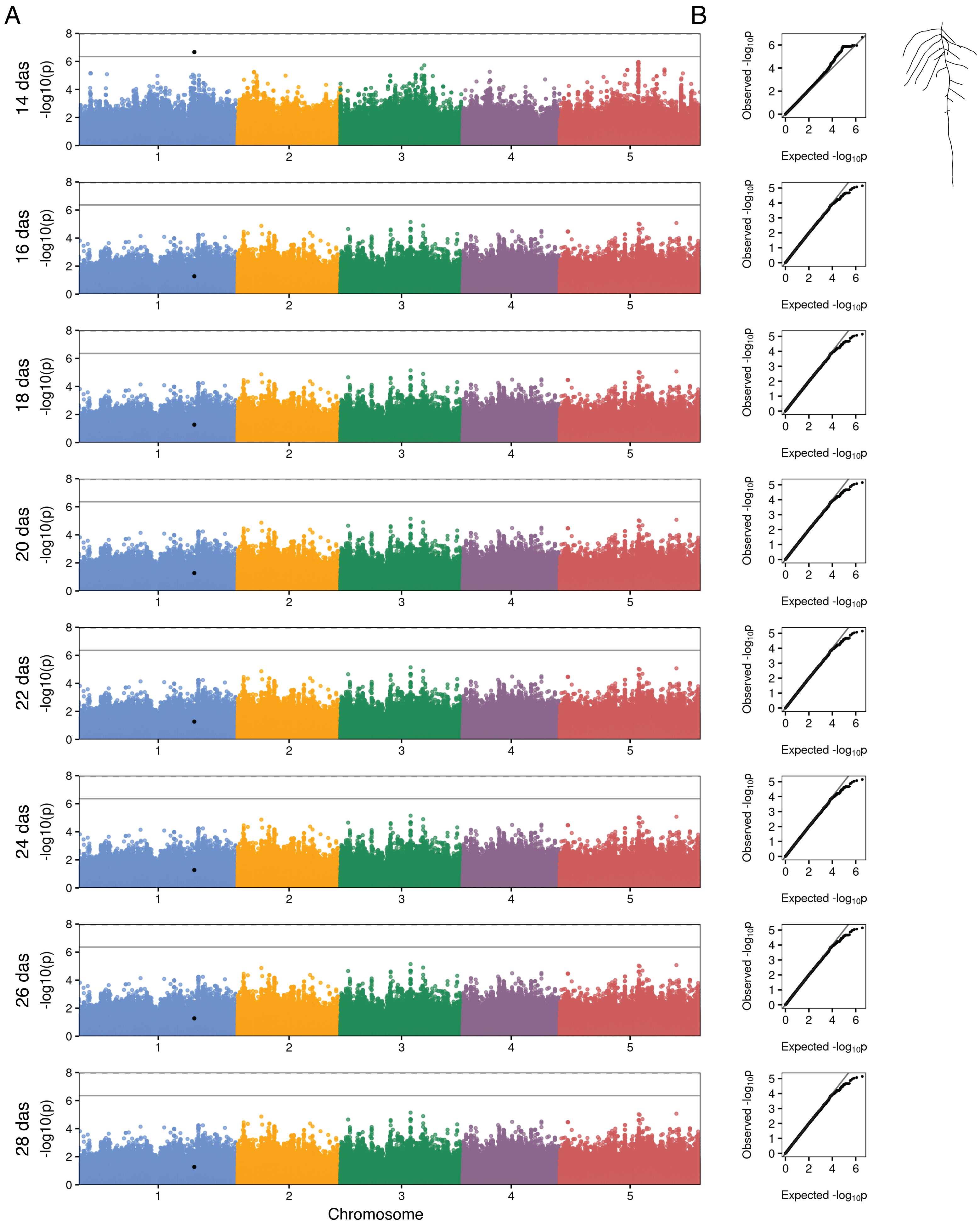

Width

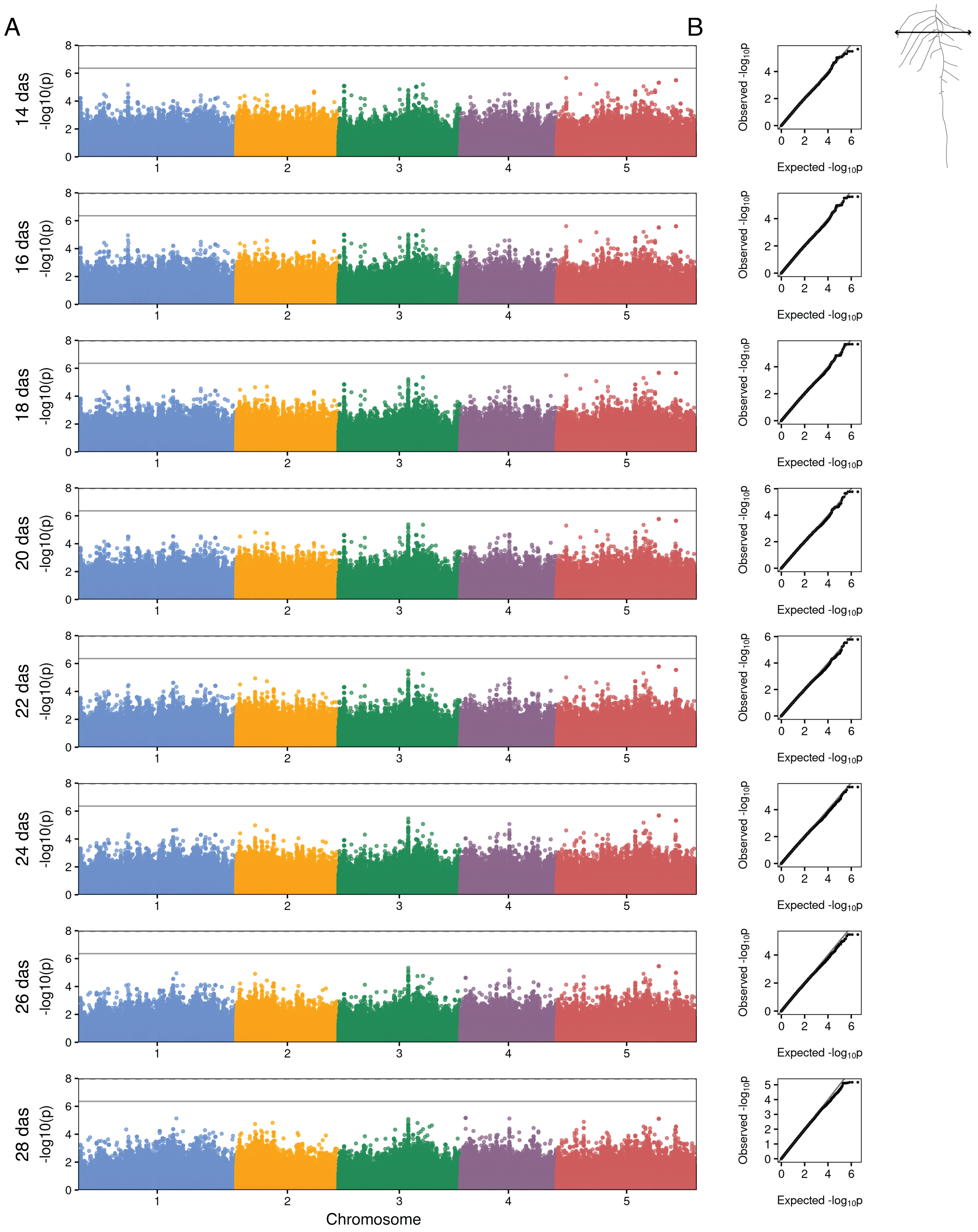
A
B
14 das
16 das
18 das
20 das
22 das
24 das
26 das
28 das
-log10(p)
Chromosome
1
2
3
4
5
Observed -log10p
Expected -log10p

Y-center

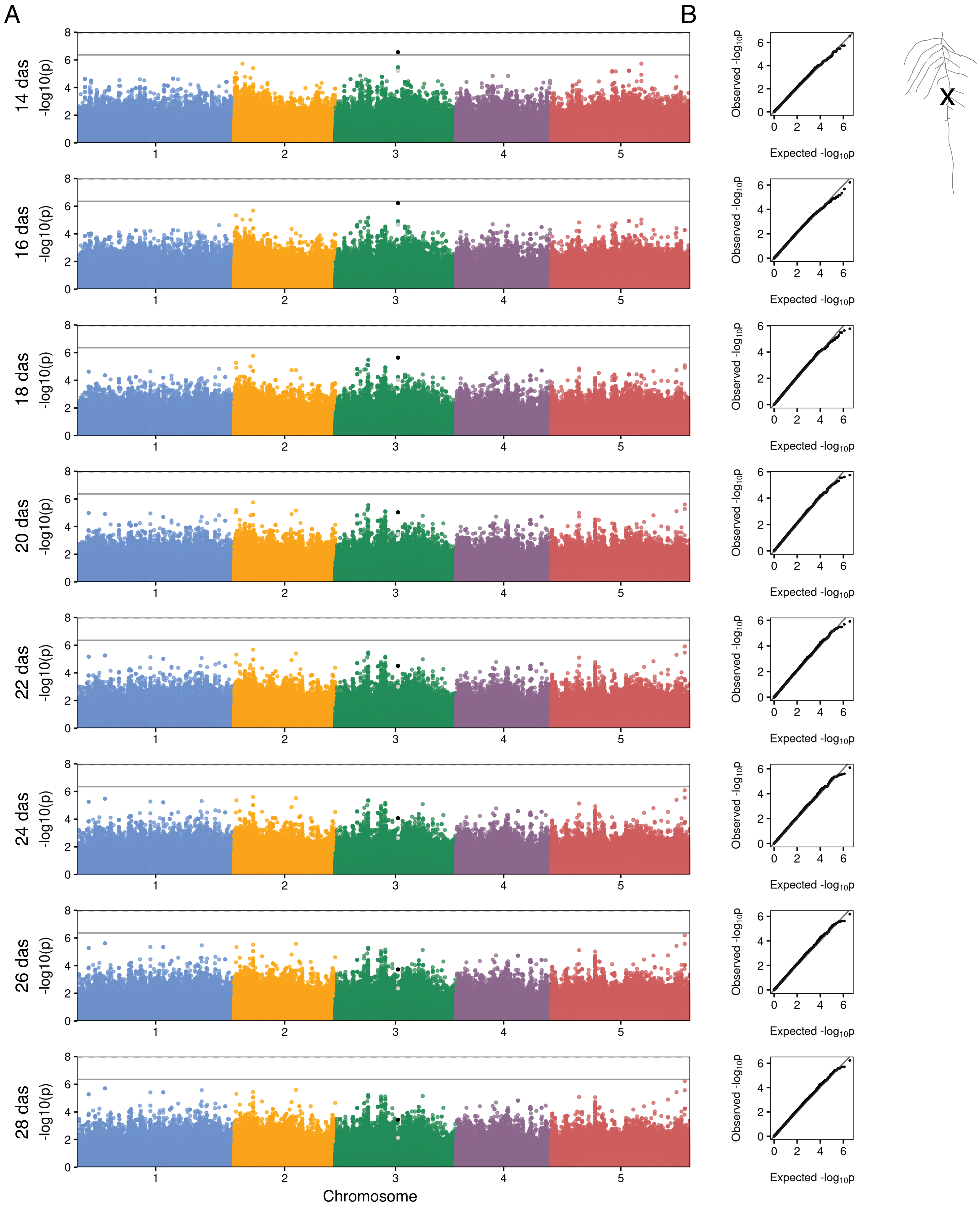

A
B
14 das
16 das
18 das
20 das
22 das
24 das
26 das
28 das
-log10(p)
Chromosome
1
2
3
4
5
Observed -log$_{10}$p
Expected -log$_{10}$p
X

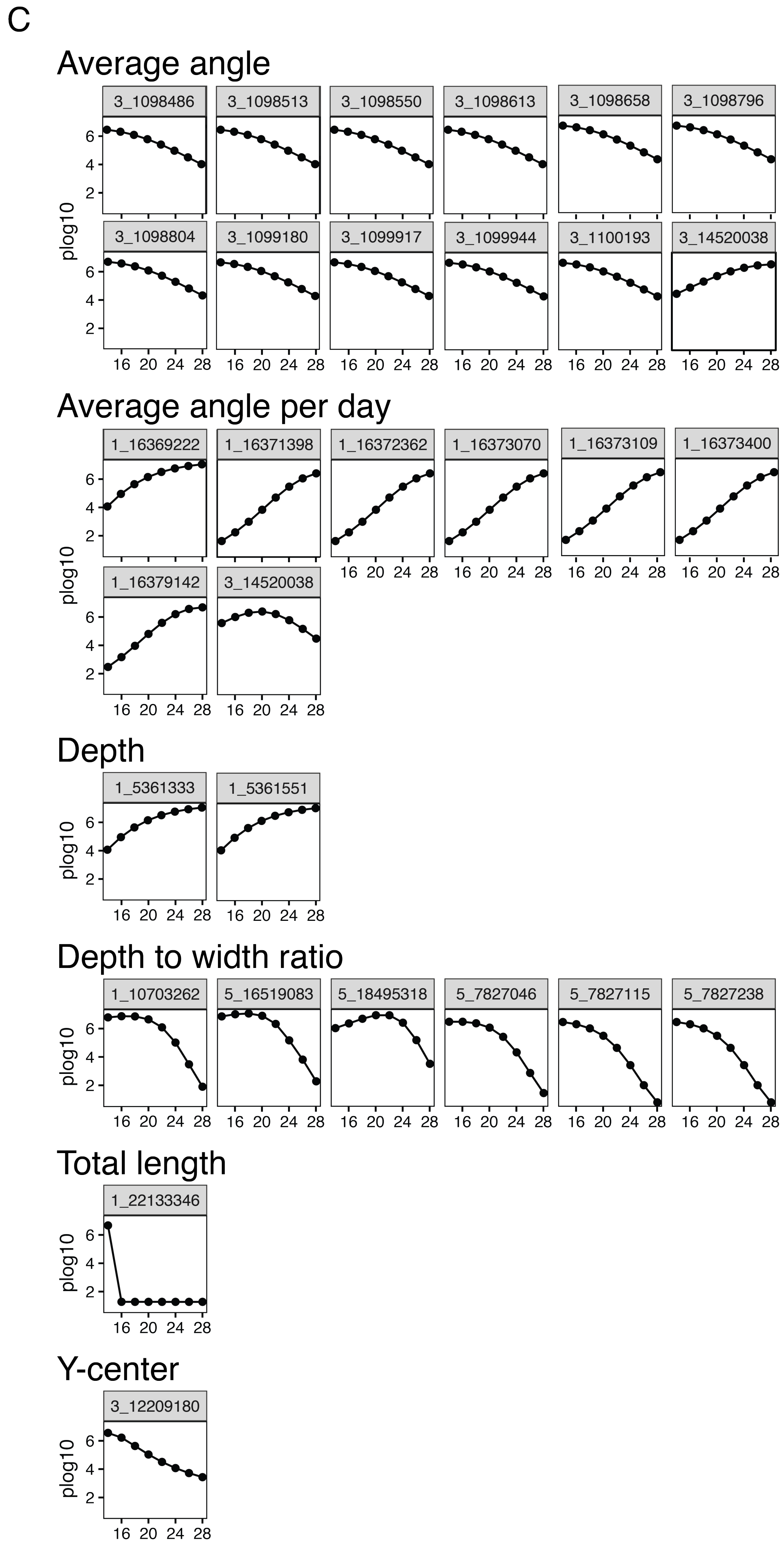
C
Average angle
3_1098486
3_1098513
3_1098550
3_1098613
3_1098658
3_1098796
3_1098804
3_1099180
3_1099917
3_1099944
3_1100193
3_14520038
Average angle per day
1_16369222
1_16371398
1_16372362
1_16373070
1_16373109
1_16373400
1_16379142
3_14520038
Depth
1_5361333
1_5361551
Depth to width ratio
1_10703262
5_16519083
5_18495318
5_7827046
5_7827115
5_7827238
Total length
1_22133346
Y-center
3_12209180
plog10
16 20 24 28
2 4 6
