# Supplementary material for: Uncovering natural variation in root system architecture and growth dynamics using a robotics-assisted phenomics platform
Source: eLife. 2022 Sep 1;11:e76968. doi: 10.7554/eLife.76968 (PMC9499532; doi:10.7554/eLife.76968)
Supplement: Transparent reporting form [file elife-76968-transrepform1.docx]

***eLife’s* transparent reporting form**

We encourage authors to provide detailed information *within their submission* to facilitate the interpretation and replication of experiments. Authors can upload supporting documentation to indicate the use of appropriate reporting guidelines for health-related research (see [EQUATOR Network](http://www.equator-network.org/%20)), life science research (see the [BioSharing Information Resource](https://biosharing.org/)), or the [ARRIVE guidelines](http://www.plosbiology.org/article/info:doi/10.1371/journal.pbio.1000412) for reporting work involving animal research. Where applicable, authors should refer to any relevant reporting standards documents in this form.

If you have any questions, please consult our Journal Policies and/or contact us: [editorial@elifesciences.org](mailto:editorial@elifesciences.org).

**Sample-size estimation**

- You should state whether an appropriate sample size was computed when the study was being designed
- You should state the statistical method of sample size computation and any required assumptions
- If no explicit power analysis was used, you should describe how you decided what sample (replicate) size (number) to use

Please outline where this information can be found within the submission (e.g., sections or figure legends), or explain why this information doesn’t apply to your submission:

Selection of accessions for the GWA was based on transformation efficiency and previous data as outlined in Supplemental Figure 4. The number of accessions used, 93, was chosen due to space availability within, and imaging capacity of, GLO-Bot. Previous studies indicated sufficient sample size to identify candidate genes in Arabidopsis. Specifically, [Rosas *et al.* 2013](https://www.pnas.org/content/110/37/15133) and [Atwell *et al.* 2010](https://pubmed.ncbi.nlm.nih.gov/20336072/) used 69 and 107 accessions, respectively, to identify candidate genes in *Arabidopsis*, showing the power to identify signals even with limited sample size.

**Replicates**

- You should report how often each experiment was performed
- You should include a definition of biological versus technical replication
- The data obtained should be provided and sufficient information should be provided to indicate the number of independent biological and/or technical replicates
- If you encountered any outliers, you should describe how these were handled
- Criteria for exclusion/inclusion of data should be clearly stated
- High-throughput sequence data should be uploaded before submission, with a private link for reviewers provided (these are available from both GEO and ArrayExpress)

Please outline where this information can be found within the submission (e.g., sections or figure legends), or explain why this information doesn’t apply to your submission:

Replicate numbers for the experiments with the six accessions can be found in the “Time-lapse imaging of six Arabidopsis accessions reveals distinct root growth trajectories” section of the main text and in the respective figure legends. Biological replicates for the diversity panel consisted of three independent transgenic lines as described in the “Biological components” section of the Materials and Methods. These biological replicates were each grown twice to comprise the six replicates mentioned in the “The GLO-Roots diversity panel” section of the main text.

**Statistical reporting**

- Statistical analysis methods should be described and justified
- Raw data should be presented in figures whenever informative to do so (typically when N per group is less than 10)
- For each experiment, you should identify the statistical tests used, exact values of N, definitions of center, methods of multiple test correction, and dispersion and precision measures (e.g., mean, median, SD, SEM, confidence intervals; and, for the major substantive results, a measure of effect size (e.g., Pearson's r, Cohen's d)
- Report exact p-values wherever possible alongside the summary statistics and 95% confidence intervals. These should be reported for all key questions and not only when the p-value is less than 0.05.

Please outline where this information can be found within the submission (e.g., sections or figure legends), or explain why this information doesn’t apply to your submission:

Statistical reporting can be found in the figure legends. Methodological approaches and statistical models used in the GWAS are described in the methods section.

(For large datasets, or papers with a very large number of statistical tests, you may upload a single table file with tests, Ns, etc., with reference to sections in the manuscript.)

**Group allocation**

- Indicate how samples were allocated into experimental groups (in the case of clinical studies, please specify allocation to treatment method); if randomization was used, please also state if restricted randomization was applied
- Indicate if masking was used during group allocation, data collection and/or data analysis

Please outline where this information can be found within the submission (e.g., sections or figure legends), or explain why this information doesn’t apply to your submission:

Samples were not divided into groups. For the diversity panel all 93 accessions were grown at the same time.

**Additional data files (“source data”)**

- We encourage you to upload relevant additional data files, such as numerical data that are represented as a graph in a figure, or as a summary table
- Where provided, these should be in the most useful format, and they can be uploaded as “Source data” files linked to a main figure or table
- Include model definition files including the full list of parameters used
- Include code used for data analysis (e.g., R, MatLab)
- Avoid stating that data files are “available upon request”

Please indicate the figures or tables for which source data files have been provided:

The root images, raw root trait data, calculated traits, fitted traits, and associated analysis scripts have all been uploaded to Zenodo and have individual DOIs.
